# Catarrh of the Bladder; Albumen in the Urine

**Published:** 1880-05

**Authors:** Henry Thompson

**Affiliations:** Surgeon Extraordinary to His Majesty the King of the Belgians; Consulting Surgeon to University College Hospital; and Emeritus Professor of Clinical Surgery


### CATARRH OF THE BLADDER; ALBUMEN IN THE URINE.

BY SIR HENRY THOMPSON,

Surgeon Extraordinary to His Majesty the King of the Belgians; Consulting Surgeon to University College Hospital; and Emeritus Professor of Clinical Surgery.

THERE is a group of symptoms frequently met with in men of advancing years, to which I desire especially to call your attention. One of the first circumstances to attract notice is that the urine is more or less cloudy when passed, and that on standing it deposits some adhesive opaque matter in the bottom of the vessel. Such urine is generally neutral, or at best faintly acid; occasionally it is alkaline, always becoming so rapidly by keeping. On interrogating the patient, you learn that the act of micturition is performed rather more frequently than natural—that he is disturbed by it two or three times in the night, and every two hours or so during the day. He may also complain of dull pains about the pelvis and back; he finds the effort to pass water rather greater than it formerly was, and the general health has of late suffered a little.

Now it is by no means uncommon to hear this group of symptoms spoken of as indicating the presence of a "catarrh of the bladder." And catarrh of the bladder is very generally regarded as a particularly obstinate, sometimes indeed as an incurable, affection. And I am free to confess that as long as those phenomena are considered referable to a specific disease, "catarrh," so long probably will the disease prove rebellious to treatment and sometimes even incurable.

Again, when the urine described is examined by the microscope, a quantity of pus varying much in different cases, is seen to be present in it; and when examined by heat and nitric acid, a certain amount of albumen is, as a matter of course, deposited.

It happens to me, in the course of consultations, to observe that these phenomena—the admixture of pus with the urine and the presence of albumen—are, singly or together, frequently regarded in themselves, and apart from other facts, as necessarily presenting indications of very grave importance. Are they so?

Certainly they are by no means necessarily grave; on the contrary, in the great majority of cases of elderly men, the presence of these products is not grave. Let me demonstrate to you the marked distinction which exists between the significance which albumen possesses in two different categories of cases. I can scarcely ask your attention to a matter of higher practical importance.

1. When a patient's urine, habitually clear, acid, and free from the faintest blood-tint, throws down to the test of heat and nitric acid a notable quantity of albumen, the source of that albumen is the renal circulation, and, if persistent, the case is almost certainly one of grave import. The presence of organic change in the kidney structure is to be inferred, and other evidence of its existence, if sought for, will probably be found.

2. A very slight admixture of blood in any urine, no matter what the source of the hemorrhage, will produce a considerable deposit of albumen. It is evident, then, that the product in such cases, although sometimes grave, is not necessarily so, and that it may furnish an indication of the slightest possible import, inasmuch as a little blood may appear in the anterior passages, from a lesion which is slight and temporary in its nature.

3. Pus in the urine may, and most commonly does, proceed from some local condition of the bladder, occasionally, indeed, from local inflammation of the urethra. Nevertheless, albumen will be deposited on applying appropriate tests. It is evident that albumen, resulting simply from pus produced by chronic cystitis, has an import vastly less grave than that described above as No. 1, being a purely local and mostly temporary affection of an organ which has no vital function, but merely a mechanical one; the albumen in the former instance being evidence of disorganization in the structure of a vital organ—that is, one the sufficient action of which is essential to the very existence of the body.

In short, there ought not to be the slightest temptation to confound two states so utterly unrelated as the two states which we have here contrasted, although they offer from one point of view an accidental similarity—that is, there is in both an admixture of albuminous material in the urine.

Still, nothing is more common than to hear, in connection with a case of purely local bladder affection, the remark gravely and significantly made, "I assure you I have, on several occasions, found by testing a large quantity of albumen in the patient's urine." It is a little difficult sometimes, although necessary, to listen to such an observation with quite sufficient patience. Does the observer really desire to intimate that the patient has constitutional albuminuria, *i. e.*, some form of Bright's disease? If not, his remark is simply devoid of meaning; since, as we know, there is vesical pus in the urine, we know equally that the albuminous constituent must appear on applying the test. And vesical pus in the urine certainly has no more relation to constitutional albuminuria than pus which comes from an external abscess or surrounds a common boil. Simple as all this may appear to you and to me, it is quite astonishing how much confusion there is in men's minds in regard to this matter, and how much importance some persons attach to all albumen found in a urinary test-tube, although the source of the deposit may be easily demonstrated to be the bladder, and no other part of the organs which lie above it.

Finally, the important practical point in relation to treatment is first to ascertain the occasion of the local catarrh. In nine out of ten of these cases it consists in inability, often only to a slight extent, on the part of the patient to empty the bladder completely. The universally acknowledged cause, hypertrophy of the prostate, is, of course, the first in order of frequency. But after this are others not infrequent. Defective action may be due, first, to simple atony, the result of past habitual or occasional over-distension of the bladder with urine; secondly, to thickened and incompetent muscular parietes of the bladder after chronic inflammation, sometimes associated with old stricture; thirdly, to defective innervation seen in connection with other slight signs of impaired function in a nervous centre; the last being, of course, the most serious of all, in its nature and probable results.

In all of these, local treatment, by carefully removing all the secretion by means of a soft catheter two or three times a day, perhaps aided by gently washing out some remainder, is the chief efficient remedy. Remember that this incompetence of the bladder is always to be sought for by physical examination; no other form of evidence in relation to it, as the patient's sensations, etc., is to be accepted as trustworthy. The introduction of a soft catheter immediately after the patient has passed water by his natural efforts, is the only test, and it should be applied on two or three occasions before arriving at a definite conclusion. The causal relation between the group of symptoms enumerated at the outset, and the defective function described, is far more common than it is generally supposed to be. It is on this account, therefore, that I have asked your attention specially to the subject.—*London Lancet, April, 1880.*

